# Contralateral Muscle Imbalances and Physiological Profile of Recreational Aerial Athletes

**DOI:** 10.3390/jfmk4030049

**Published:** 2019-07-25

**Authors:** Rachel M. Ruggieri, Pablo B. Costa

**Affiliations:** Exercise Physiology Laboratory, Department of Kinesiology, California State University, Fullerton, CA 92831, USA

**Keywords:** circus artists, circus acts, aerial silks, aerial hoop, aerial fitness, acrobats

## Abstract

Background: Aerial fitness is quickly gaining popularity; however, little is known regarding the physiological demands of aerial athletes. The purpose of the study was to examine contralateral muscle imbalances, compare dominant versus non-dominant hamstrings-to-quadriceps (H:Q) ratios, and to establish a physiological profile of recreational aerial athletes. Methods: Thirteen aerialist women visited a local aerial studio to participate in a data collection session to examine isometric levels of upper and lower body strength, muscle endurance, flexibility, balance, and cardiovascular fitness. Results: No significant differences were found between dominant and non-dominant hand grip strength (*p* = 0.077), dominant and non-dominant isometric knee flexion (*p* = 0.483), dominant and non-dominant isometric knee extension (*p* = 0.152), or dominant and non-dominant isometric H:Q ratios (*p* = 0.102). In addition, no significant difference was found between isometric dominant H:Q ratio and the widely-used value of 0.60 (*p* = 0.139). However, isometric non-dominant H:Q ratio was significantly lower than the 0.60 criterion (*p* = 0.004). Aerial athletes demonstrated to have excellent flexibility, balance, cardiorespiratory fitness, and average strength. Conclusions: Aerial fitness may be another recreational activity that could be used to maintain higher levels of flexibility, balance, cardiorespiratory fitness, and strength. Aerialists may want to consider focusing on strengthening the lower body and balancing the hamstrings and quadriceps muscle strength.

## 1. Introduction

**Figure 1:**
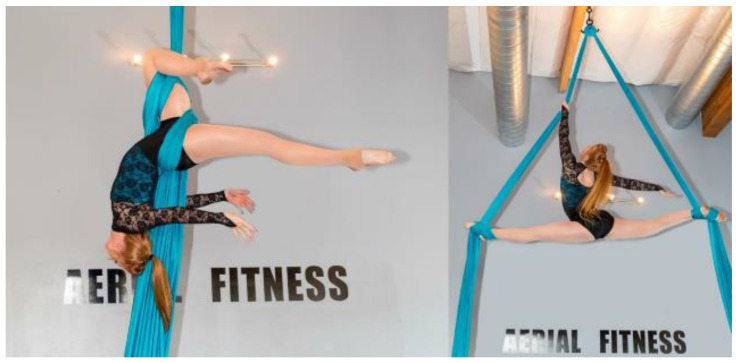
Aerial artist performing on the silks apparatus.

Aerial is an activity performed suspended in the air while hanging on various apparatuses such as hoops and silks [1,2] (Figure 1). Aerial athletes demonstrate strength, flexibility, endurance, and coordination, all while airborne [1,2]. The activity of aerial arts is mainly associated with shows and circus arts, and was once considered to be unique [1,2]. In recent years, aerial has been gaining popularity as a fitness activity to be performed recreationally. Hence, aerial is quickly becoming a more regular form of exercise for maintaining fitness [1,2].

Previous physiological and anthropometric profiles have been conducted on athletes with similar performance demands, such as dancers and gymnasts, who all execute difficult and complex routines similar to aerialists [1,2]. These athletes require strength, flexibility, balance, and precision in short explosive bursts, which set them apart from other sports [3,4,5]. Although many studies have been dedicated to understanding the physical fitness levels of dancers and gymnasts, little is known regarding the fitness levels of aerial athletes. 

Participating in aerial arts could potentially lead to injury given the likely demands of the activity, and what is known from other sports with similar demands (e.g., dance and gymnastics) [1,2,6]. While lower body injuries are common across all athletic populations, non-contact sports-related injuries associated with strength and muscle imbalance are commonly influenced by the hamstrings-to-quadriceps (H:Q) ratios [7]. Weak hamstrings, or hamstrings dominated by quadriceps strength, increase the likelihood of injury [7,8]. Injuries such as anterior cruciate ligament tears and hamstrings strains can occur when quadriceps maximal torque is produced, and when the hamstrings do not generate equivalent counter force to decelerate rotation in movements when the knee is being extended [8]. Findings have shown isometric or conventional H:Q ratios below 0.60 increase injury risk in many types of athletes [9,10], and the lower extremities in circus athletes and acrobats are the most commonly sprained and strained region in the body in these activities [11].

Physiological data currently not in existence is needed to further understand the physical airborne demands of aerial athletes. Physiological variables, such as strength, flexibility, balance, cardiorespiratory fitness, body composition, and H:Q ratios, collected on aerial athletes could help determine their fitness levels compared to various athletes such as dancers and gymnasts, and could predict injury risk in the sport. In addition, a physiological profile could be beneficial in further understanding the workload of aerial arts, specifically to prepare and enhance training to excel in the activity. Therefore, the purpose of this study was to establish a physiological profile of recreational aerial athletes. In addition, another aim of the study was to examine the athletes’ H:Q ratios to report data not currently available, and to compare risk of lower body injuries between dominant and non-dominant limbs. 

## 2. Materials and Methods 

### 2.1. Subjects

Thirteen aerialist women (mean age ± SD = 32.8 ± 6.3 years; height = 161.7 ± 5.8 cm; body mass = 129.6 ± 13.8 lbs; BMI = 22.5 ± 2.6 kg·m^−2^) volunteered to participate in the study. All participants had trained in the aerial arts at least twice a week for the past six months, and were determined to be recreationally active by a health and physical activity questionnaire. 10 out of 13 subjects reported engaging in aerobic fitness 4.9 ± 3.0 h per week, 3 reported engaging in resistance training 3.0 ± 1.0 h per week, and all 13 reported to participate in a recreational sport (aerial fitness) 6.0 ± 3.4 h per week. Aerialists typically trained two to five times per week, for approximately one to three hours per day. Training normally involves a warm-up and stretching for 30 min prior to touching any apparatus. Then, aerialists typically move on to conditioning on their apparatus of choice. After conditioning, aerialists will carry out moves involving extreme flexibility or strength, or other moves involving wraps, drops, or knots. Participants were advised to not exercise before testing, and testing sessions were conducted approximately in the same time during the afternoon. In addition, all participants were free of current injury, although three participants were uncomfortable with completing the non-dominant leg testing due to past hamstrings discomfort. Prior to starting the session, an Informed Consent form was read and signed by all participants. The study (HSR-18-19-55) was approved by the CSUF Institutional Review Board on 14 December 2018. 

### 2.2. Procedures

Subjects visited a local aerial studio to participate in one data collection session. The participants’ anthropometrics were recorded first. Height and body mass were measured using a scale and stadiometer (438, Detecto, Webb City, MO, USA) to calculate body mass index (BMI). Body fat percentage was predicted using skinfolds with a seven-site protocol. Skinfold sites, measured by an experienced evaluator, included triceps, subscapular, axillary, chest, abdominal, suprailliac, and thigh, measured to the nearest millimeter (mm) using a skinfold caliper (SFC-1000, Harpenden, Burgess Hill, England) (coefficient of variation (CV) = 18%, intraclass correlation (ICC) = 0.94) [12,13]. 

Hand grip strength was measured for both the dominant and non-dominant hand using a hand dynamometer (5030L1, Lafayette, Lafayette, IN, USA). The dynamometer’s handle was adjusted to fit the heel of the palm, and the middle of the four fingers for each participant. Participants were instructed to keep their arm down by their side and squeeze as hard as possible for five seconds. Each side was measured three times, and the highest score was recorded in kg (CV dominant hand grip = 17%, non-dominant hand grip = 18%, ICC = 0.90) [12,13,14].

Back strength was measured with a back strength dynamometer (5002, Takei, Niigata, Japan). Participants were instructed to step onto the dynamometer, grab the bar, and pull as hard as possible for three seconds. The bar was adjusted to each participant’s height, to be above knees and across the thighs. Participant’s trunk remained upright with the knees flexed. Participants held the bar using a pronated grip. The highest of three measurements was recorded in kg (CV = 22%) [12].

Leg strength was measured with a commercially available dynamometer designed for field testing (5002, Takei, Niigata, Japan). For knee extension, participants sat in a chair with their testing leg at 90°. Participants were instructed to kick out with maximal effort for three seconds. The highest of three trials was measured in kg and recorded. For knee flexion, participants laid on the floor in a prone position with their testing leg at 90°. Participants were instructed to pull in with maximal effort for three seconds. The highest of three trials was measured in kg and recorded. H:Q ratios were determined by dividing hamstrings isometric peak force by quadriceps isometric peak force (CV dominant knee extension = 28%, non-dominant knee extension = 35%, dominant knee flexion = 42%, non-dominant knee flexion = 37%, dominant H:Q ratio = 37%, non-dominant H:Q ratio = 44%) [15].

Upper body endurance was measured using a pull-up test. Participants hung from an aerial hoop and performed as many pull-ups as possible in one trial. Pull-ups were performed with an overhand grip, with no kicking or swinging. The participant’s chin had to clearly pass over the top of the bar. Pull-ups performed incorrectly were not counted (CV = 83%) [16].

A standard sit-and-reach test was administered to measure lower back and hamstrings flexibility. Participants were instructed to place their bare feet flat against the sit-and-reach box (12-1085, Baseline, White Plains, NY, USA), to maintain knees straight, and to place their arms out with the palms facing down. Participants were allowed three practice trials, keeping their hands level with each other as they reached forward as far as possible. Participant’s fourth sit-and-reach length was recorded to the nearest cm (CV = 13%) [12].

Multiple hip and shoulder joint range of motion (ROM) sites were measured with a goniometer (12-1001, Baseline, White Plains, NY, USA) to determine upper and lower body flexibility. Hamstrings muscle length, hip flexion, extension, abduction, and adduction were measured, as were shoulder flexion, extension, abduction, and adduction in degrees by the experienced evaluator. All range of motion protocols were followed as described by Berryman Reese and Bandy [17]. (CV hip flexion = 9%, hamstring muscle length = 8%, hip extension = 25%, hip abduction = 15%, hip adduction = 13%, shoulder flexion = 2%, shoulder extension = 24%, shoulder abduction = 0.3%, shoulder adduction = 16%, ICC shoulder flexion 0.98, shoulder extension 0.94, shoulder abduction 0.95, hip flexion 0.94, hip extension 0.82, hip abduction 0.90, hip adduction 0.90, shoulder adduction 0.59) [17,18].

A balance test was administered to measure static balance. Participants removed their shoes and were instructed to stand on one foot with their hands crossed over their chest. Participants were told to place the non-dominant foot in front of the ankle of the dominant foot, and to focus on one spot while balancing. This was performed once with the eyes open, and once with eyes closed. The balance test was terminated if the participant’s arms uncrossed, or were moved to assist their balance, if either foot moved, if closed eyes were to open, or if the test exceeded 45 s. Participants were instructed to practice the balanced posed before being recorded to the nearest millisecond (CV unipedal stance eyes open = 0%, unipedal stance eyes closed = 91%, balance test-retest reliability eyes closed = 0.74, and eyes open = 0.91) [12].

The last test administered was a Queens College Step Test. The three-minute step test was used to estimate cardiovascular endurance. Participants stepped to a cadence of 22 steps per minute on a 16.25” bench step. After the three minutes, participant’s heart rate was measured and recorded after 15 seconds using a heart rate monitor (FT1, Polar, Bethpage, NY, USA). The recovery heart rate was used to determine participant’s predicted cardiovascular endurance as VO_2max_ (CV = 6%) [12,14].

### 2.3. Statistics

Means and standard deviations for all variables were calculated. Four dependent t-tests were used to compare hand grip, leg flexion, leg extension strength, and H:Q ratios between dominant and non-dominant limbs. Two one-sample *t*-tests were used to compare dominant and non-dominant H:Q ratios to the widely used value of 0.60. Effect sizes (ES) based on Cohen’s [19] were used to compare difference, where 0.2 represented a small difference, 0.5 represented a moderate difference, and 0.8+ represented a large difference. IBM SPSS Statistics version 25 (IBM Corp., Armonk, NY, USA) was used for all statistical analyses. An α-level of *p* ≤ 0.05 was considered statistically significant for all comparisons.

## 3. Results

**Figure 2:**
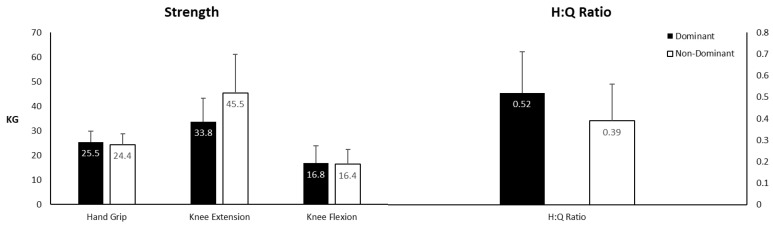
Means ± SD for strength values and hamstrings-to-quadriceps (H:Q) ratios of dominant and non-dominant limbs.

Table 1 displays means (±SDs) and ranges for all variables. Figure 2 displays means and SDs for the hand grip, knee extension, knee flexion, and H:Q ratio data. There were no significant differences between dominant and non-dominant hand grip (*p* = 0.077, ES = 0.256 (small)), dominant and non-dominant knee flexion (*p* = 0.483, ES = 0.061 (moderate)), dominant and non-dominant knee extension (*p* = 0.152, ES = 0.862 (large)), or H:Q ratios (*p* = 0.102, ES = 0.687 (moderate)). No significant difference was found between dominant H:Q ratios and the widely used value of 0.60 (*p* = 0.139). However, there was a significant difference between non-dominant H:Q ratios and the 0.60 criterion. Non-dominant H:Q ratios were significantly lower than the 0.60 value (*p* = 0.004). 

## 4. Discussion

This study presents insight on recreational aerial athletes, and establishes their physiological profile. The aerialists’ body fat % (BF%) presented in this investigation are similar to athletes found in a previous study [3]. Professional female dancers of classical ballet and modern dance styles have shown to have BF% ranging from 13.0–26.9% [3]. Thus, the BF% presented by aerialists in the present investigation fall within the range of the professional dancers. Novak et al. [20] reported collegiate female dancers from various dance style programs had similar BF% to the aerialists in the current study (20.5 ± 4.6 %), and to be significantly leaner than their control group (26.5 ± 3.6). The control group did not participate in any sort of physical activity, concluding both the dancers and the aerialists in the current study had a lower BF% than a sedentary population [20]. In addition, a study conducted on gymnasts reported teenage female athletes had a BF% of 15.3 ± 3.3 [21]. In comparison to the aerialists, the gymnasts had a lower BF%, although age differences between the gymnasts (16.9 ± 3.6 years) and the aerialists (32.8 ± 6.3 years) should be taken into consideration [21]. In contrast, an investigation on female collegiate gymnasts found the athletes to have BF% of 23.2 ± 3.2, which are higher than the BF% of aerialists determined in the present investigation [22]. Differences in BF% between athletes are likely due to the nature of their physical activity [21].

VO_2max_ previously displayed by collegiate female dancers of various dance styles (41.5 ± 6.7 mL·k^−1^·min^−1^) between the ages of 19–29 exhibited findings very similar to those of the aerial athletes [20]. In contrast, VO_2max_ of gymnasts between 13.5 ± 1.27 years of age was found to be higher (47.9 ± 4.0 mL·k^−1^·min^−1^) than the aerialists presented in this study [23]. Although the gymnasts presented with a higher VO_2max_ than the aerialists, age must be taken into consideration, since VO_2max_ has been shown to decline with age [24]. This decline is due to a number of factors including a decrease in maximum heart rate, decreased peripheral blood flow, decreased stroke volume, and a decrease in both vital capacity and forced expiratory volume [25]. Many studies have found differences in VO_2max_ among different styles and performance level of dancing. When performance status is taken into consideration, principle dancers were shown to have the highest VO_2max_ when compared to corps de ballet, soloist, and 1st artists [26]. For dance style comparisons, VO_2max_ differences were found when comparing Standard, Latin, and Ten dance styles [27]. For VO_2max_, Latin dance was found to have the highest (53.6 ± 5.4 mL·k^−1^·min^−1^), with Standard dance style in the middle (51.8 ± 4.6 mL·k^−1^·min^−1^), and Ten dance having the lowest (50.0 ± 7.6 mL·k^−1^·min^−1^) [27]. The differences in VO_2max_ for each style is likely due to the various demands of each Dancesport style; however, all Dancesport styles had an average VO_2max_ higher than the present VO_2max_ of the aerialist in the present study [27]. Nevertheless, separating the aerialists in the current study by apparatus was not taken into consideration. The athletes were grouped together regardless of use of apparatus, which is a possible limitation of the present investigation. Future studies should examine whether VO_2max_ of aerial athletes vary by apparatus, performance status, or age groups. Ten of the 13 aerialists also reported 4.9 ± 3.0 h per week of aerobic exercise. While it is not clear which precise activity resulted in the excellent VO_2max_, aerial fitness would provide benefits that an isolated aerobic training session would not, such as improvements in balance, flexibility, muscle endurance, and strength.

With lower extremity injuries being prevalent across athletic populations [28], it is important to assess muscle imbalances to prevent these injuries [28]. Approximately 87% of work injuries have been shown to happen to acrobats (tumbling/floor acrobatics, trapeze, and tightrope artists) in a gym setting, with the lower extremities most commonly affected in women [11]. H:Q ratio is frequently used to predict the risk of lower body injuries, with isometric or conventional H:Q ratios below 0.60 being a common indicator of muscle imbalances [8,9,29]. The aerialists in the present study did not meet the 0.60 cut off for the non-dominant leg. In a study performed on collegiate female gymnasts, the percentage of gymnasts that did not meet the 0.60 criterion for concentric conventional H:Q ratio was 69% at 60°·s^−1^ as well 77% at 120°·s^−1^ [29]. When the H:Q ratios of ballet dancers were compared to modern dancers, it was found ballet dancers had slightly lower knee extension strength, slightly higher knee flexion strength, and higher H:Q ratios than the modern dancers at 180°·s^−1^ [30]. This suggests H:Q ratio imbalances may be style- or sport-specific, as different sporting activities alter strength characteristics [30,31,32]. Training programs of athletes with higher H:Q ratios could be used as a reference to develop injury prevention and strength training programs for athletes with low H:Q ratios. Future studies should compare the H:Q ratios on aerialists from different performance apparatuses, or how implementing different training programs could affect the H:Q ratios in aerial athletes. Out of the 13 subjects in the current study, only 10 could complete the H:Q ratio testing on the non-dominant side due to past hamstrings discomfort, which could possibly be a limitation of this study. This limitation could potentially influence the significant difference between the non-dominant isometric H:Q ratios and the 0.60 criterion value.

Hand grip has previously been used to predict muscle strength and endurance, and increased hand grip strength is associated with increased upper body strength [33,34]. Gymnasts require a high grip strength to be able to perform well in their events, and aerial athletes require some level of grip strength to perform on their apparatuses as well [2,33]. When compared to female gymnasts, the aerialists presented with an average, but 66% lower grip strength [35]. Gymnasts were found to have excellent grip strength, with a right hand grip strength of 45 ± 5.0 kg, and a left hand grip strength of 38 ± 4.0 kg [35]. In relation to muscle endurance, female gymnasts ages 6–18 years were found to have an average of 8.3 ± 4.2 pull-ups for a day 1 test, and an average of 8.6 ± 3.4 pull-ups for a day 2 test [4], while young male gymnasts with an average age of 11.0 ± 1.0 years were shown to be able to perform 13.1 ± 6.1 pull-ups [36]. Both the young male and female gymnasts performed a higher number of pull-ups than the aerialists presented in the current study, but were also much younger and performed the pull-ups on a stationary bar whereas the present study used the aerial hoop for accessibility and specificity. In contrast, ballet dancers across all ages typically are shown to have poor upper body strength [5]. The aerialists also displayed a wide range of pull-ups, which might contribute to the high CV%. While there are no official categories for aerial levels, subjects had different amounts of training experience and training volume. Considering the aerial athletes displayed average strength, aerial fitness may be considered a recreational activity to be performed regularly to maintain upper body strength and endurance.

High levels of ROM and flexibility are essential in activities such as aerial, gymnastics, and ballet dancing, and are generally found to be greater because of the performance demands from the activities [2,5,21,37,38]. Professional, ballet, and contemporary dancers overall displayed significantly greater ranges of flexibility over control groups [3,39], while competitive dancers presented with increased hamstrings flexibility compared to recreational dancers [38]. Gymnasts also displayed greater flexibility than other athletes as well [21]. George et al. [21], reported the female athletes had a sit-and-reach flexibility of 36.4 ± 4.2 cm, which means the aerialists in the current study had a sit-and-reach flexibility 18% higher than gymnasts. Ballet dancers were reported to have a sit-and-reach flexibility of 22.8 ± 4.1 cm, which means the aerialists in the current study had a 48% higher sit-and-reach flexibility score [40]. Participating in aerial fitness may be a notable recreational activity to increase flexibility. However, care must be considered to balance the flexibility requirements of the aerial arts activity and the potentially negative effects when stretching is performed before exercise [41,42]. Static stretching can affect muscle strength and performance, as well as H:Q ratios, increasing the risk of lower extremity injuries [41,42].

Static and dynamic balance are both a vital component of motor skills for executing complex skills in sports settings [43]. Gymnasts and ballet dancers are thought to have exceptional balance as a result of their sport [38,43]. In the present study aerial athletes demonstrated excellent balance as well. As a result, aerial arts may be pursued as a fitness activity to increase balance. Analyses of balance have determined modern dancers had better static and dynamic balance than active non-dancers [44]. Recreational ballet dancers, who only participated in ballet classes, were found to have better dynamic balance than competition dancers, who participated in ballet classes as well as competitions [38]. In contrast, gymnasts have been found to have superior static and dynamic balance compared to soccer and basketball players and swimmers [37,43,44]. In the present investigation, one subject held the closed eyes balance test for the complete 45.1, becoming an outlier, causing the wide range for the balance variables, and possibly causing the large CV%.

## 5. Conclusions

No significant differences were found between dominant and non-dominant hand grip strength, dominant and non-dominant flexion, extension, and H:Q ratios, or between dominant H:Q ratios and the minimum suggested ratio to avoid hamstrings and knee-related injuries. Significant differences were only found between the athlete’s non-dominant H:Q ratios and the widely used 0.60 value. In addition, recreational aerial athletes appear to have excellent flexibility, balance, cardiorespiratory fitness, and average strength according to normative data [12,13]. This study presents the first documentation on recreational aerial athletes, which could play a role in further understanding the fitness levels of this activity, may support aerial arts as a healthy recreational fitness pursuit, and may contribute to recommending the appropriate exercise prescription for athletes of this sport. 

Aerial athletes have been shown to have similarities to both gymnasts and dancers [3,5,20,21,29,37,44]. These variables of cardiorespiratory endurance, body composition, H:Q ratios, strength, flexibility, and balance can be used in future studies to further analyze the demands of aerial athletics, perhaps with training interventions. These variables can also be used to develop training programs to enhance aerial performance, and reduce the risk of lower extremity and contralateral limb injury in the sport of aerial. Sports-related injuries associated with strength and muscle imbalances are commonly influenced by the H:Q ratios [7,8]. Aerialists were shown to have average strength and may want to consider focusing on lower body strength training targeted to specifically strengthen the hamstrings and balance the H:Q ratios.

## Figures and Tables

**Table 1:** Physiological Profile Means ± SD and (Ranges) of Recreational Aerial Athletes.

*N* = 13	Strength		Flexibility		Balance and Body Fat Percentage	Predicted VO_2max_ (mL·k^−1^·min^−1^)
Dominant Hand Grip (kg)	25.5 ± 4.3 (20–36)	Sit and Reach (cm)	44.5 ± 6.0 (30–52)	Unipedal Balance Eyes Open (sec)	45.1 ± 0 (45.1)	41.8 ± 2.4 (38.47–45.12)
Non-Dominant Hand Grip (kg)	24.4 ± 4.3 (20–32)	Hip Flexion (°)	128.2 ± 11.4 (113–145)	Unipedal Balance Eyes Closed (sec)	13.7 ± 12.5 (3.4–45.1)	
Back (kg)	83.7 ± 18.0 (60–115)	Hamstring Muscle Length (°)	108.5 ± 8.5 (94–125)	Skinfolds Body Fat (%)	18.4 ± 3.4	
Dominant Knee Extension (kg)	33.8 ± 9.4 (20–50)	Hip Extension (°)	33.5 ± 8.4 (20–46)			
Non-Dominant Knee Extension (kg)	45.5 ± 15.7 (24–71)	Hip Abduction (°)	45.2 ± 6.7 (34–55)			
Dominant Knee Flexion (kg)	16.8 ± 7.1 (10–35)	Hip Adduction (°)	113.6 ± 14.7 (82–150)			
Non-Dominant Knee Flexion (kg)	16.4 ± 6.1 (9–27)	Shoulder Flexion (°)	178.8 ± 3 (170–180)			
Dominant H:Q Ratio (kg)	0.52 ± 0.19 (0.25–0.88)	Shoulder Extension (°)	63.8 ± 15.4 (39–90)			
Non-Dominant H:Q Ratio (kg)	0.39 ± 0.17 (0.23–0.72)	Shoulder Abduction (°)	179.8 ± 0.6 (178–180)			
Pull-ups	4.56 ± 3.81 (0–12)	Shoulder Adduction (°)	64.5 ± 10.4 (51–83)			
